# Evaluation of ion recombination and polarity effect on photon depth dose measurements using mini‐ and micro‐ion chamber

**DOI:** 10.1002/acm2.14495

**Published:** 2024-11-02

**Authors:** Antonella Fogliata, Andrea Bresolin, Pasqualina Gallo, Francesco La Fauci, Marco Pelizzoli, Giacomo Reggiori, Luca Cozzi

**Affiliations:** ^1^ Radiotherapy and Radiosurgery Dept Humanitas IRCCS Research Hospital and Cancer Center Milan‐Rozzano Italy

**Keywords:** FFF, Ion recombination factor, photon beams, polarity effect

## Abstract

**Purpose:**

To investigate the effect of ion recombination ($k_{s}$) and polarity ($k_{pol}$) correction factors on percentage depth dose (PDD) curves for three ion chambers, using flat and flattening filter free (FFF) beams, across different broad field sizes. A method to assess these effects and their corresponding corrections is proposed.

**Methods:**

$k_{s}$ and $k_{pol}$ were evaluated following the IAEA TRS‐398 protocol for three ion chambers: PTW Semiflex‐3D‐31021, PinPoint‐3D‐31022, and Semiflex‐31010. PDD measurements were acquired from d_max_ to 32 cm depth at four voltages (± 400 V or ± 300 V, and ± 100 V) for field sizes 4 × 4, 10 × 10, 20 × 20, and 40 × 40 cm^2^. The $k_{s}$ values were computed after the $k_{pol}$ correction at each applied voltage. This study aimed to assess the variation of the factors along scanning depths for different field sizes, to evaluate the need for correcting the scanning data.

**Results:**

$k_{pol}$ was independent of depth and field size for Semiflex‐3D, while it presented an increasing value with depth for the PinPoint‐3D. $k_{s}$ increased with dose rate, i.e., decreased with depth. The variation of this perturbation effect over the PDD range was about 0.1% for flat beams with all three ion chambers. With FFF beams, it was around 0.3% with PinPoint‐3D, and 0.8% for 10FFF with Semiflex‐3D. A second‐order polynomial fit can be determined to directly correct the raw data scanned along the beam axis for both $k_{s}$ and $k_{pol}$.

**Conclusion:**

$k_{s}$ can significantly affect the PDD scanning measurements in FFF beams acquired with Semiflex‐3D. An error close to 1% at large depths could be present, meaning an error of less than 0.3% relative to the dose at the depth of d_max_.

## 1 INTRODUCTION

Ion recombination and polarity corrections have been extensively studied in the context of reference dosimetry in clinical photon beams. With the emergence and increased use of flattening filter free (FFF) beams, there has been a growing interest in evaluating the perturbation parameters, particularly ion recombination ($k_{s}$) and polarity ($k_{pol}$) effects, not only in reference conditions but also during scanning data measurements.^1^, ^2^, ^3^, ^4^, ^5^, ^6^, ^7^, ^8^

The dependence of $k_{s}$ on the dose per pulse (DPP) has been well established.^9^, ^10^, ^11^, ^12^ Published studies have shown its variation over a wide range of DPP by varying the beam energy, from flattened to FFF beams. FFF photon beams have higher DPP than flattened beams, resulting in larger $k_{s}$.

The DPP also varies with depth and off‐axis position, leading to possible dose rate variation of $k_{s}$ with depth in a percentage depth dose (PDD) or with off‐axis position in a profile scan.

Small chambers, such as mini‐chambers (∼0.1 cm^3^ volume) or micro‐chambers (∼0.01 cm^3^ volume), are commonly used for scanning beam data acquisitions, with ion chambers being considered the gold standard for scanning measurements. These chambers, with their reduced perturbation volume effect, allow reasonable estimation of the penumbra and are applicable in all dose gradient conditions, including FFF beam profiles.

Some studies have shown that small ion chambers can exhibit anomalous polarity and ion recombination effects.^13^, ^14^ Published literature reports the variation of $k_{s}$ with Jaffé plots (relating the inverse of the detector reading to the inverse of the applied voltage) and the estimation of the variation of ion recombination factor as a function of DPP.^2^, ^8^, ^9^, ^11^ The evaluation of the $k_{s}$ variation with depth is generally neglected in scanning measurements in common practice, possibly due to the rather short range of the DPP variation in the same scan, leading to limited, although present, variation of the ion recombination effect. It is, however, a known potential issue, as also pointed out for PDDs by Corns et al.^7^ and, recently, Kojima et al.^8^ However, both studies estimated only the ion recombination correction, not including the polarity effect in the final possible correction.

This study aims to assess the changes in $k_{s}$ and $k_{pol}$ across PDDs generated by flattened and FFF beams under various conditions using three specific ion chambers, Semiflex‐3D, PinPoint‐3D, and Semiflex (PTW, Freiburg, Germany). Additionally, it aims to develop a straightforward method for analyzing the depth‐dependent response. The main goal is to provide a tool for understanding the suitability of selecting an ion chamber for linac commissioning measurements and, if necessary, providing guidance on addressing both $k_{s}$ and $k_{pol}$ effects together for practical correction factors use, particularly in FFF beams.

## 2 MATERIALS AND METHODS

### 2.1 Ion recombination correction factor

The two‐voltage method for determining the ion recombination effect, as recommended in the IAEA TRS‐398 code of practice,^15^ has been validated against the more robust Jaffé plot method.^2^, ^16^, ^17^ In this study, the ion recombination correction factor, $k_{s}$, is estimated using the two‐voltage formula from the TRS‐398:

(1) $$\;k_{s}=a_{0}\;+a_{1}\left(\frac{M_{1}}{M_{2}}\right)+a_{2}{\left(\frac{M_{1}}{M_{2}}\right)}^{2}$$

where $M_{1}$ and $M_{2}$ are the readings obtained at two voltages, $V_{1}$ and $V_{2}$, respectively. The coefficients $a_{n}$ are taken from Table 4.VII of the TRS‐398. The ratio of the two voltages $\frac{V_{1}}{V_{2}}$, is recommended to be ≥3 in the TRS‐398.

### 2.2 Polarity effect correction factor

The polarity correction factor, $\;k_{pol}$, is determined according to the TRS‐398 as follows:

(2) $$\;k_{pol}=\left(\frac{\left|M_{+}\right|+\left|M_{-}\right|}{2M}\right)\;$$

where $M_{+}$ and $M_{-}$ are the reading at positive and negative polarity, respectively, and M is the reading at the working polarity. The equation indicates that the true value is the average of the readings with positive and negative polarity.

### 2.3 Tested ion chambers

Two ion chambers have been thoroughly analyzed in this work: a Semiflex‐3D and a PinPoint‐3D. A third ion chamber, a Semiflex, was used to test a proposed procedure. The detectors’ specifications are summarized in Table 1. All three ion chambers were positioned with the stem perpendicular to the beam's central axis (horizontal position) during measurements.

**TABLE 1:** Specifications of the used PTW ion chambers.

	Semiflex‐3D	PinPoint‐3D	Semiflex
Type	31021	31022	31010
Radius of sensitive volume	2.4 mm	1.45 mm	2.75 mm
Length of sensitive volume	4.8 mm	2.9 mm	6.5 mm
Nominal sensitive volume	0.07 cm^3^	0.016 cm^3^	0.125 cm^3^
Wall of sensitive volume material	PMMA, graphite	PMMA, graphite	PMMA, graphite
Central electrode material	Aluminium	Aluminium	Aluminium
Nominal chamber voltage	+400 V	+300 V	+400 V

### 2.4 Beam and measurement settings

Four beam energies from a Varian TrueBeam linear accelerator (Varian Medical Systems, Palo Alto, California, USA) have been analyzed: 6 and 10 MV flattened (6X and 10X) and FFF (6FFF and 10FFF). The nominal dose per pulse (reported at d_max_, 100 cm of source to surface distance, SSD) are 0.28, 0.28, 0.78, and 1.31 mGy/pulse for the above energies, respectively, operating at their maximum pulse repetition rate of 600 MU/min for the flattened beams and 1400 and 2400 MU/min for the 6 and 10 FFF. The reference dose calibration condition of the used TrueBeam is 1 Gy/100 MU ad d_max_ for SSD = 100 cm.

For each detector and each beam quality, PDDs at SSD = 90 cm (as commonly used during linac commissioning), from d_max_ down to 32 cm depth, have been acquired at four different voltages: +400, −400, +100, −100 V for the Semiflex‐3D and +300, −300, +100, −100 V for the PinPoint‐3D. These acquisitions allowed the determination of $k_{pol}$ for each voltage and $k_{s}$ using the two‐voltage method. Four square field sizes: 4 × 4, 10 × 10, 20 × 20, and 40 × 40 cm^2^ were acquired and studied. Stabilization of the chamber at each polarizing voltage setting was assured with pre‐irradiation. All measurements were acquired at the maximum pulse repetition rate available for each energy, as typically done during scanning measurements.

A PTW BeamScan water phantom was used with its built‐in reference class electrometer. A step‐by‐step scanning mode, with 2 cm point separation and a measurement collection time of 1 s per point, was adopted for all beam qualities and the detectors. No reference chamber was used. For each acquisition, three measurements were performed, and the average value was used for the analysis, along with its standard deviation of the mean as measurement uncertainty.

### 2.5 Estimation of the correction factors $k_{s}$ and $k_{pol}$

The $k_{pol}$ was determined, according to Equation 2, for each of the applied voltages *V_1_* and *V_2_*. This allowed the estimation of the $k_{s}$ correction factor with Equation 1 based on measured values at the two voltages corrected for the polarity effect, including possible $k_{pol}$ variation with depth or applied voltage that can influence the determination of the $k_{s}$ at each condition.

In the following, $k_{s}$ refers to the $k_{s}$ corrected for $k_{pol}$ at each specific applied voltage and measurement condition. The possible dependence of $k_{s}$ on depth (or dose) and field size was determined for the four beam qualities.

### 2.6 Estimation of the uncorrected PDD error and output factor error

The ratio between the uncorrected (at +400 or +300 V depending on the detector) and $k_{s}$ corrected PDDs was determined to estimate the error in PDD measurements for each detector and beam quality during a common scanning acquisition at a single voltage setting without correction for polarity or ion recombination effects.

The $k_{s}$ values have also been evaluated at a fixed depth of 10 cm for the different field sizes to quantify the possible error when using these detectors for output factor measurements in broad beams, determining the output factor as the ratio of the two detector readings between the test and reference field sizes.

### 2.7 Statistical error

Type A uncertainty was obtained from the propagation of errors, using the deviation of each repeated measurement defined as the standard error of the mean. The type A uncertainty was considered dominant in this work, and no other uncertainties were included.

### 2.8 Suggested procedure to estimate ion chamber accuracy

A schematic workflow to determine the ion chamber accuracy in terms of combined $k_{s}$ and $k_{pol}$ is proposed to easily estimate the accuracy of PDDs during, for example, a linac commissioning data acquisition.

Procedure steps:

1. At the working SSD and used beam quality, acquire PDDs:
  - For the representative and a large field sizes (10 × 10 and 40 × 40 cm^2^),
  - With large stepping (3 – 5 cm),
  - With long enough collection time (∼1 s)
  - At four different voltages, e.g., +400, +100, −400, −100 V (the high voltage should be the working one, and the low voltage should be chosen to have a ratio with the high voltage of 3 or 4 to apply the TRS‐398 two‐voltage formula, Equation 1, and its parameters)
2. At each depth, apply Equation 1 for $k_{s}$ determination, where the measured data $M_{1}$ and $M_{2}$ are the readings corrected for the polarity influence quantity, i.e., the average between the positive and negative polarity for each voltage, in each condition.
3. Estimate the correction factor at each depth as the ratio between $k_{s}$ corrected PDD data (combined $k_{s}$ and $k_{pol}$) and uncorrected data
4. The depth‐dependent correction factor is obtained by fitting a second‐order polynomial to the results at each depth determined in step 3. This result can help decide on using a depth‐dependent correction.

To prove and test the procedure, a different detector was used, a PTW Semiflex, type 31010, ion chamber, whose characteristics are summarized in Table 1. The results of this test, in addition to proving the procedure, provide information on an additional ion chamber.

## 3 RESULTS

### 3.1 Polarity correction factors $k_{pol}$

**FIGURE 1:**
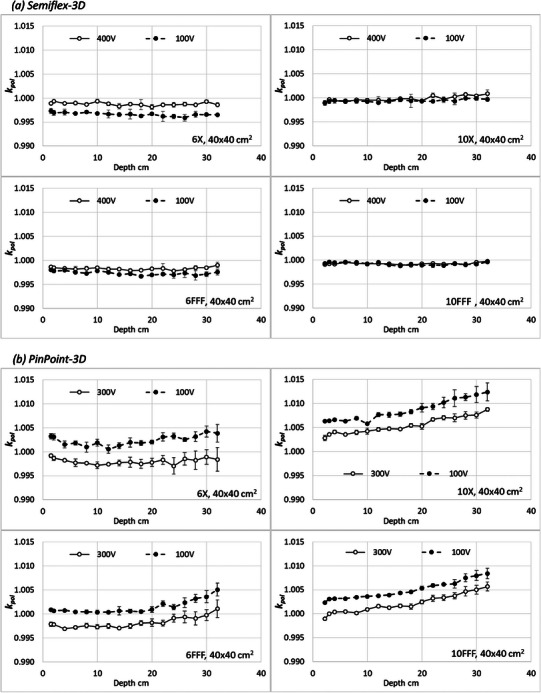
$k_{pol}$ as a function of relative dose for 40 × 40 cm^2^ field size for all the analyzed energies: (a) Semiflex‐3D, (b) PinPoint‐3D. The scale on the y‐axis is the same in all plots.

Figure 1 presents the polarity correction factors $k_{pol}$ as a function of depth for the 40 × 40 cm^2^ field, which exhibits the largest variability, under different beam qualities and applied voltages for the two fully analyzed chambers. The Semiflex‐3D chamber demonstrates stability in the $k_{pol}$ values across various conditions. In contrast, the PinPoint‐3D detector shows a trend of increasing polarity effect with depth for 10 MV beams. Notably, the variation in $k_{pol}$ values at different polarities for the same field size conditions suggests the inclusion of the $k_{pol}$ factor in the $k_{s}$ correction factor due to its influence on the recombination effect estimation using the two‐voltage method. Supplementary material includes similar plots for all field sizes and energy conditions in Figure S1.

### 3.2 Ion recombination correction factors $k_{s}$

**FIGURE 2:**
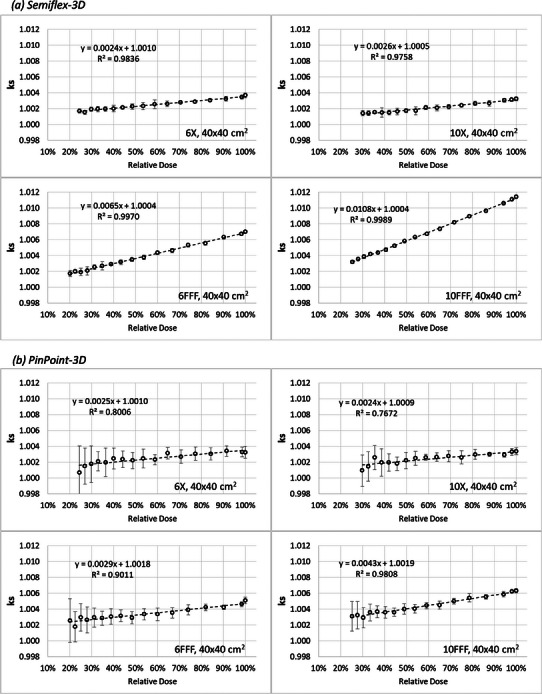
$k_{s}$ as a function of relative dose for 40 × 40 cm^2^ field size for all the analyzed energies: (a) Semiflex‐3D, (b) PinPoint‐3D. The scale on the y‐axis is the same in all plots.

The $k_{s}$ correction factors, corrected for $k_{pol}$ in each voltage condition, exhibit decreasing values with increasing depth from d_max_ to 32 cm (i.e., increasing with dose, as expected with increasing DPP). Figure 2 translates the depth dependence into relative dose dependence (normalized to 100% at d_max_), highlighting its linear pattern for the 40 × 40 cm^2^ field and four beam quality. Supplementary material includes similar plots for all field sizes and energy conditions in Figure S2.

The Semiflex‐3D chamber presents a larger variation of $k_{s}$ with dose (or depth) for FFF beams, particularly for higher FFF beam quality. In contrast, the PinPoint‐3D detector shows similar variation with the dose for unflattened beams and is not significantly dependent on beam quality or FFF modality.

Table 2 reports the percentage $k_{s}$ increase for each 10% dose increase in a PDD, obtained from the slope of the linear fit of the data shown in Figures 2 and S2 of the Supplementary material, for all field sizes. The $k_{s}$ variation with depth is constant over the range of different field sizes.

**TABLE 2:** $k_{s}$ percentage increase for each 10% dose increase in a PDD.

Detector	Field size cm x cm	6X	10X	6FFF	10FFF
Semiflex‐3D	4 × 4	0.02 ± 0.01 %	0.02 ± 0.01 %	0.06 ± 0.01 %	0.10 ± 0.01 %
	10 × 10	0.02 ± 0.01 %	0.03 ± 0.01 %	0.06 ± 0.01 %	0.10 ± 0.01 %
	20 × 20	0.03 ± 0.01 %	0.03 ± 0.01 %	0.06 ± 0.01 %	0.11 ± 0.01 %
	40 × 40	0.02 ± 0.01 %	0.03 ± 0.01 %	0.07 ± 0.01 %	0.11 ± 0.01 %
PinPoint‐3D	4 × 4	0.02 ± 0.03 %	0.02 ± 0.02 %	0.03 ± 0.02 %	0.04 ± 0.01 %
	10 × 10	0.02 ± 0.04 %	0.02 ± 0.02 %	0.03 ± 0.01 %	0.04 ± 0.01 %
	20 × 20	0.02 ± 0.04 %	0.02 ± 0.03 %	0.03 ± 0.02 %	0.04 ± 0.01 %
	40 × 40	0.03 ± 0.03 %	0.02 ± 0.03 %	0.03 ± 0.02 %	0.04 ± 0.01 %

The error is the uncertainty of the slope in the linear fit of $k_{s}$ with relative dose.

**FIGURE 3:**
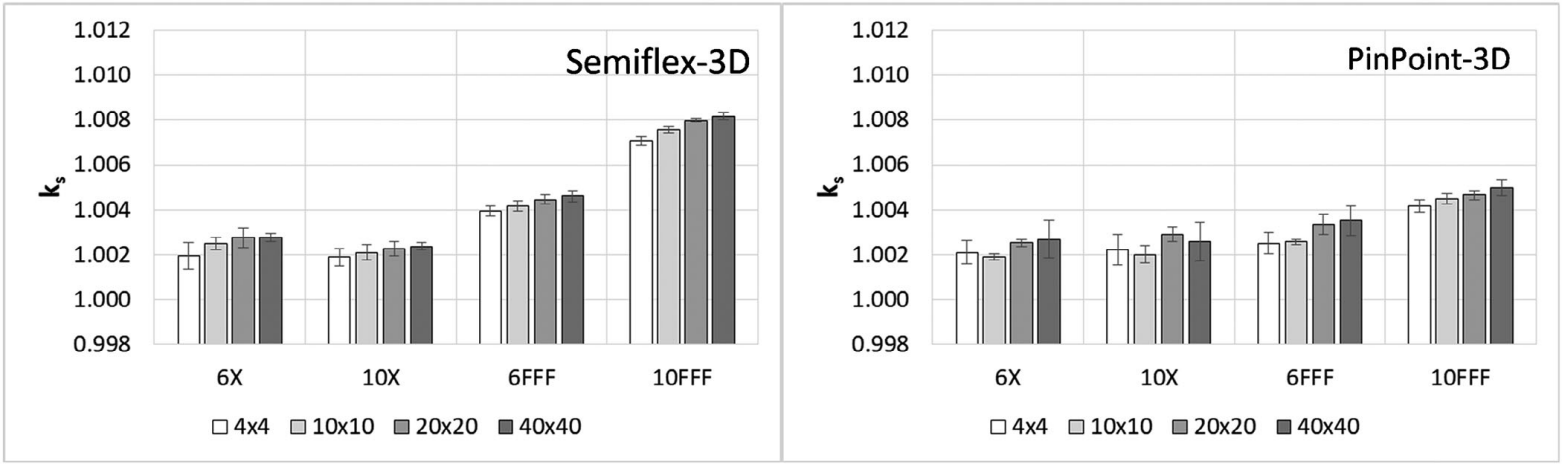
$k_{s}$ for different field sizes, at 10 cm depth. On the left: Semiflex‐3D, on the right: PinPoint‐3D. The scale on the y‐axis is the same as in Figure 2, for visualization consistency.

Figure 3 presents the values of the resulting $k_{s}$ for different field sizes from data at 10 cm depth, showing a very limited variation with field size at a fixed depth. This result confirms the suitability of both ion chambers for output factor measurements determined as the ratio of readings.

### 3.3 Uncertainty in PDD scanning measurements and depth dependence correction factor

During PDD scanning measurements, the $k_{s}$ and $k_{pol}$ are generally not accounted for, being considered as constant and not varying with depth.

**FIGURE 4:**
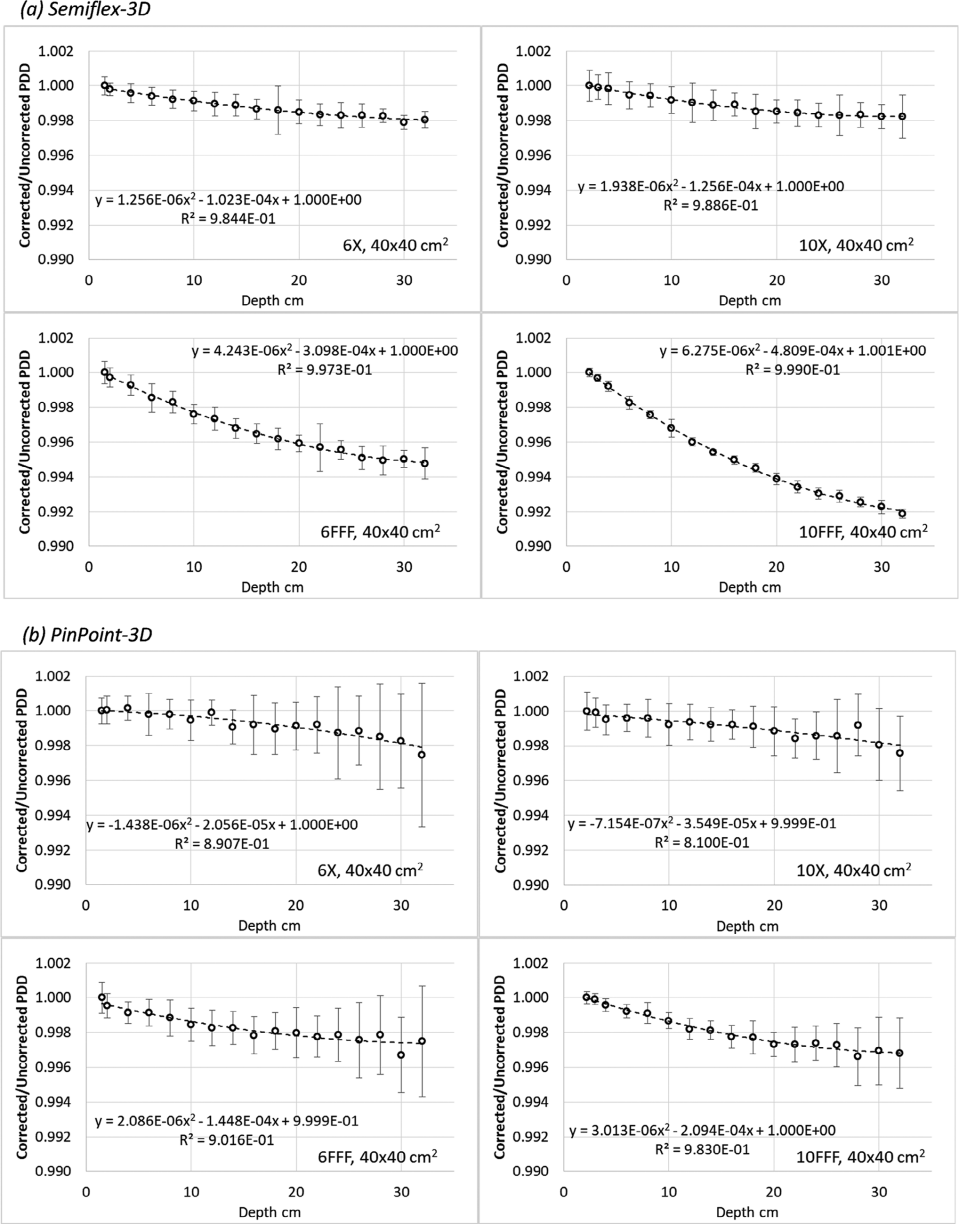
Ratio between corrected and uncorrected PDD for the 40 × 40 cm^2^ field size for all the analyzed energies: (a) Semiflex‐3D, (b) PinPoint‐3D. The fitting curve can be the correction factor to apply to the raw PDD scanned data acquired with the specific detector.

Figure 4 reports the ratios, for the 40 × 40 cm^2^ field, of PDDs from d_max_ to 32 cm depth with and without $k_{s}$ correction, both normalized to d_max_, for the four energies and two detectors. The same plots for all field sizes are reported in the Supplementary material (Figure S3). The largest variation of the correction factor was 0.8% for the 40 × 40 cm^2^ field from a 10FFF beam with the Semiflex‐3D, and 0.3% for the same beam with the PinPoint‐3D detector.

On the plots, the second‐order polynomial fit is shown, which can be directly applied to the raw PDD acquisition in the specific condition if considered relevant.

### 3.4 Determination of the depth‐dependent correction fit

**FIGURE 5:**
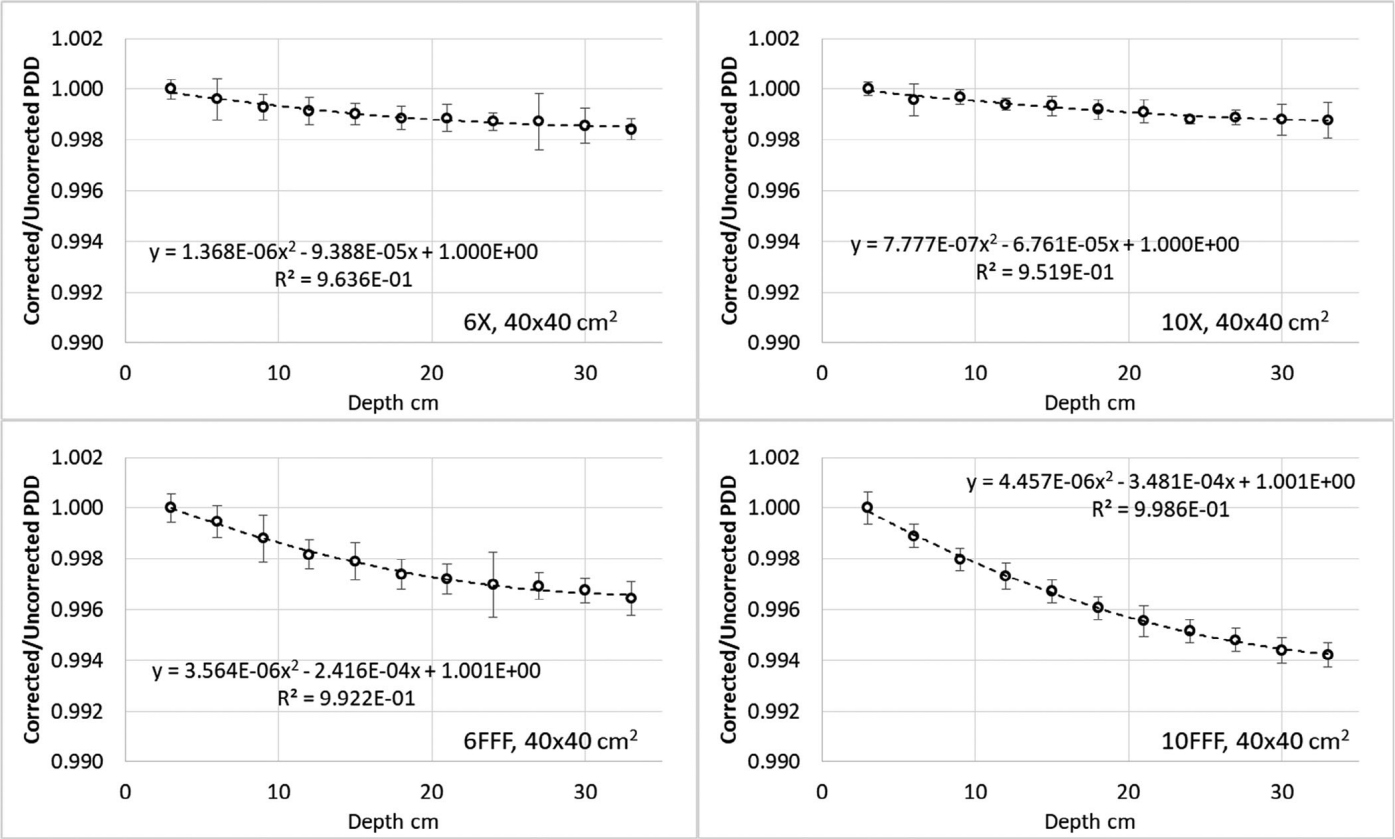
Ratio between corrected and uncorrected PDD for the 40 × 40 cm^2^ field size for all the analyzed energies for the Semiflex 0.125 cm^3^ ion chamber. The fitting curve can be the correction factor to apply to the raw PDD scanned data.

Following the procedure suggested in the Materials and Methods section for the depth dependence correction factor estimation, a Semiflex (0.125 cm^3^) ion chamber was analyzed for two field sizes (10 × 10 and 40 × 40 cm^2^). Figure S4 of the Supplementary materials presents the $k_{pol}$ as a function of depth and the $k_{s}$ as a function of the relative dose. The polarity effect is independent of depth, and a small dependence on the applied voltage is shown; the $k_{s}$ (corrected for $k_{pol}$) presents a variation with depth for large FFF beams, with the largest difference at ∼30 cm depth of ∼0.6% for a 40 × 40 cm^2^ 10FFF beam. Figure 5 shows the ratio between corrected and uncorrected PDDs for the 40 × 40 cm^2^ field; the same for the 10 × 10 cm^2^ field is reported in Figure S4 of the Supplementary materials. This ratio is the correction factor to eventually apply to the PDD scanning measurements, according to the second‐order polynomial fit parameters overlaid on the plot.

## 4 DISCUSSION

Several studies have extensively investigated the effects of polarity and ion recombination in single points, primarily focusing on reference dosimetry with various ion chambers.^1^, ^2^, ^3^, ^4^, ^5^, ^6^, ^9^, ^10^, ^11^ However, the accuracy of relative dosimetry in PDD scanning measurements has received less attention.

In this work, we investigated three commonly available ion chambers, the mini‐ion chambers Semiflex‐3D (type 31021) and Semiflex (type 31010) and the micro‐ion chamber PinPoint‐3D (type 31022), from PTW, to estimate polarity and ion recombination effects in single scanning measurement conditions.

Previous studies have reported that ion recombination increases with dose per pulse by varying the dose per pulse using different beams, from approximately 0.3 mGy/pulse in a 6X to 2.7 mGy/pulse in a 10FFF beam (Bruggmoser et al.^9^). The Semiflex‐3D showed a larger dependence than the PinPoint‐3D, with a total variation in the dose/pulse range exceeding 1.5% compared to less than 1.0%. Similar results were reported by Martin‐Martin et al.^11^ for the Semiflex‐3D. Martin‐Martin et al.^12^ also highlighted the problem of applying the ion recombination factor on PDD measurements in FFF beams, emphasizing the need to correct for the ratio of the 10 cm and d_max_ points, each with its correction factor. The authors found an inaccuracy greater than 0.5% when the $k_{s}$ factor is neglected in FFF PDD, with the 10FFF beam being the worst case. Corns et al.^7^ and Kojima et al.^8^ evaluated the $k_{s}$ over the entire PDD depth range, but their depth‐dependent correction factors did not include the $k_{pol}$ effect. Corns et al. studied a single ion chamber, Exradin A19, and found a $k_{s}$ variation over the PDD of 0.4% for FFF beams. Kojima et al. studied the Semiflex‐3D type 31021, presenting the PDD with ion recombination correction as deviation from the results of a Farmer chamber; the ion recombination correction factor variation over the PDD depth range was approximately 0.8%, slightly different for positive or negative applied polarity.

The findings of this study are consistent with those reported in previous publications. Notably, the inaccuracy in the PDD measurement at a depth of 30 cm for a large 10FFF beam using a Semiflex‐3D ion chamber without correction is nearly 0.8% (relative). Unlike published literature, this study highlights the importance of correcting for the polarity effect in all the measurements involved in the determination of recombination at different applied voltages. The possible difference in the polarity effect at, for example, 400 and 100 V suggests the inclusion of this effect in the recombination correction factor estimation with the two‐voltage method recommended by the TRS‐398.

This involves straightforward measurements at two voltages and both polarities, which can then be used to calculate a combined $k_{s}$ and $k_{pol}$ correction to apply at any depth. The result of this analysis can inform the decision to apply such a correction or to consciously accept the inaccuracy under the given conditions. In particular, the second‐order polynomial fit can be used as a correction factor as a function of depth.

Given the good agreement of the results obtained in this work with those published by other groups, a generalization of the depth dependence correction factors for the specific detector might be foreseen for more accurate measurements. A limitation of this work is the lack of a systematic evaluation of the interchamber variability, which, if small, could suggest a possible generalized applicability of the depth‐dependent correction factors for a specific detector type.

Another limitation of the study is the evaluation of only three ion chambers (two with full presentation of the results, one as proof of concept of the methodology). A more comprehensive study including other detectors could be advisable.

## 5 CONCLUSION

The three analyzed ion chambers presented $k_{s}$ or $k_{pol}$ dependence with depth during scanning PDD acquisitions. In particular, the PinPoint‐3D 31022 has a more relevant $k_{pol}$ effect, while the Semiflex‐3D 31021 presents a systematic variation of $k_{s}$ with depth, especially for FFF beams, which might require a depth‐dependent correction.

## AUTHOR CONTRIBUTIONS

**Antonella Fogliata**: Conceptualization; methodology; supervision; data acquisition and validation; investigation; writing—original draft; writing—review & editing. **Andrea Bresolin**: Data Acquisition and validation; investigation; writing—review & editing. **Pasqualina Gallo**: Data acquisition and validation; investigation; writing—review & editing. **Francesco La Fauci**: Data acquisition and validation; investigation; writing—review & editing. **Marco Pelizzoli**: Data acquisition and validation; investigation; writing—review & editing. **Giacomo Reggiori**: Data acquisition and validation; investigation; writing—review & editing. **Luca Cozzi**: Conceptualization; methodology; supervision; data acquisition and validation; investigation; writing—original draft; writing—review & editing.

## CONFLICT OF INTEREST STATEMENT

The authors declare no conflicts of interest.

## Supporting information

SUPPORTING INFORMATION
