# Supplementary material for: Evaluation of ion recombination and polarity effect on photon depth dose measurements using mini‐ and micro‐ion chamber
Source: J Appl Clin Med Phys. 2024 Nov 2;26(1):e14495. doi: 10.1002/acm2.14495 (PMC11712343; doi:10.1002/acm2.14495)

Supplementary Material of:

Evaluation of ion recombination and polarity effect on photon depth dose measurements using mini- and micro-ion chamber

Figure 1s: k_{pol} as a function of depth for 4x4, 10x10 and 20x20 cm^2 field size for all the analysed energies. The scale on the *y*-axis is the same in all plots. This Figure completes the data of Figure 1 in the main text, which reports the same results for the 40x40 cm^2 only.

(a) Semiflex-3D

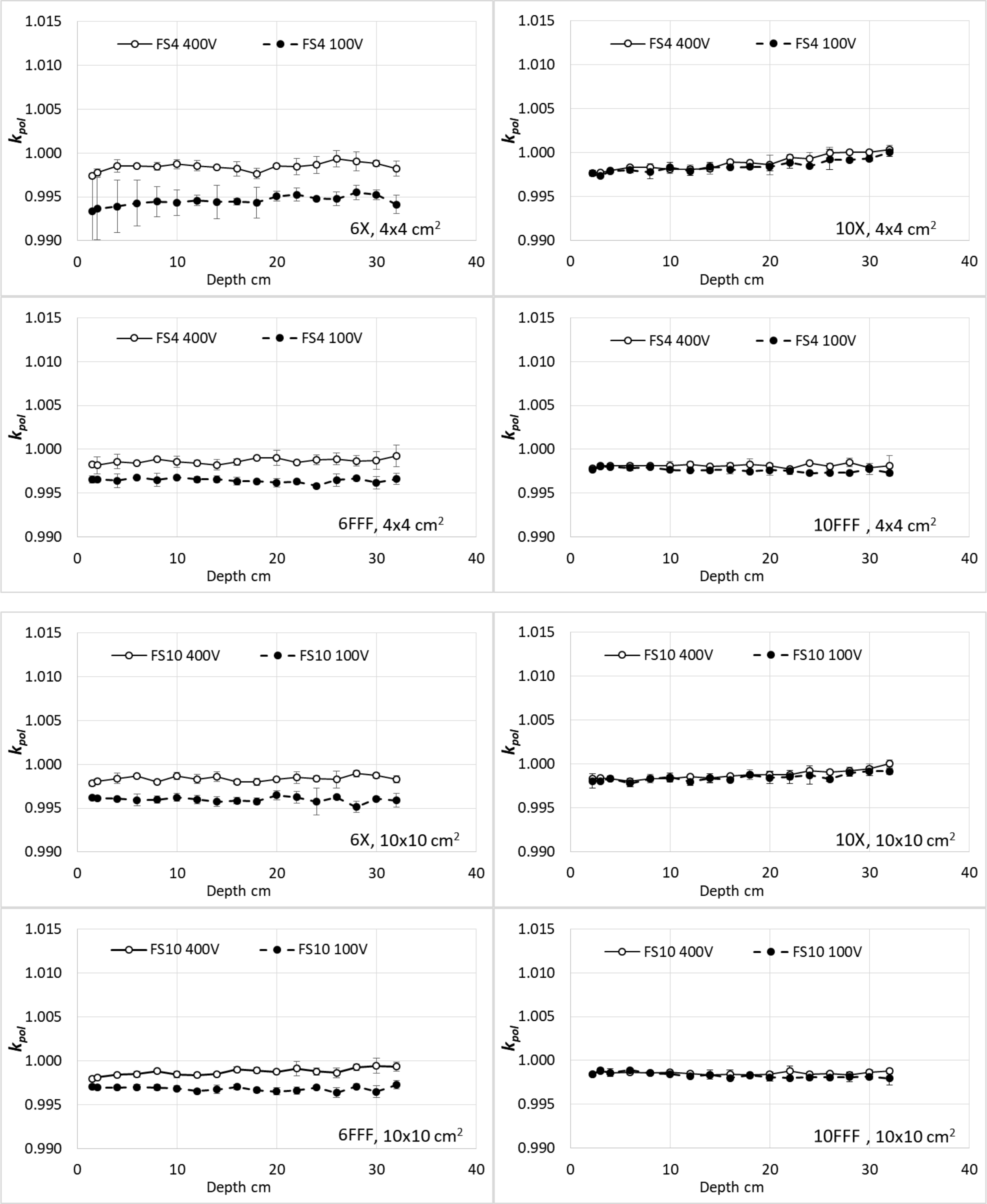

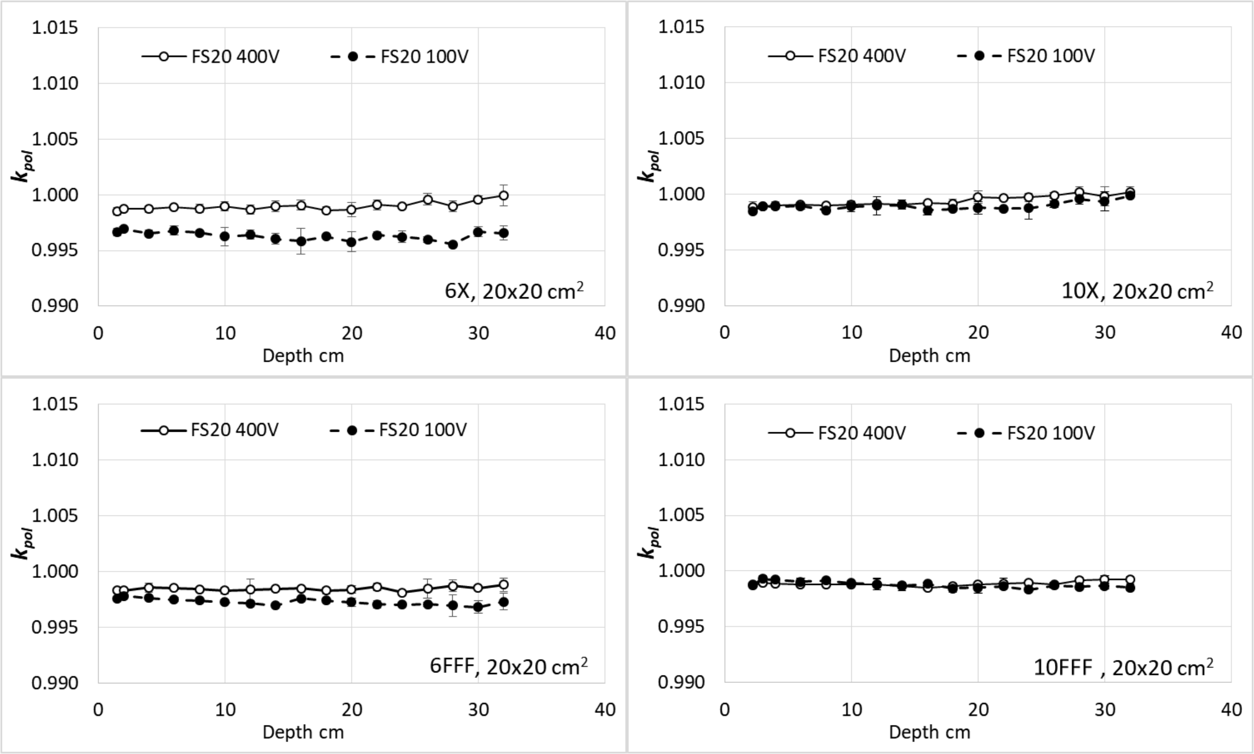

(b) PinPoint-3D

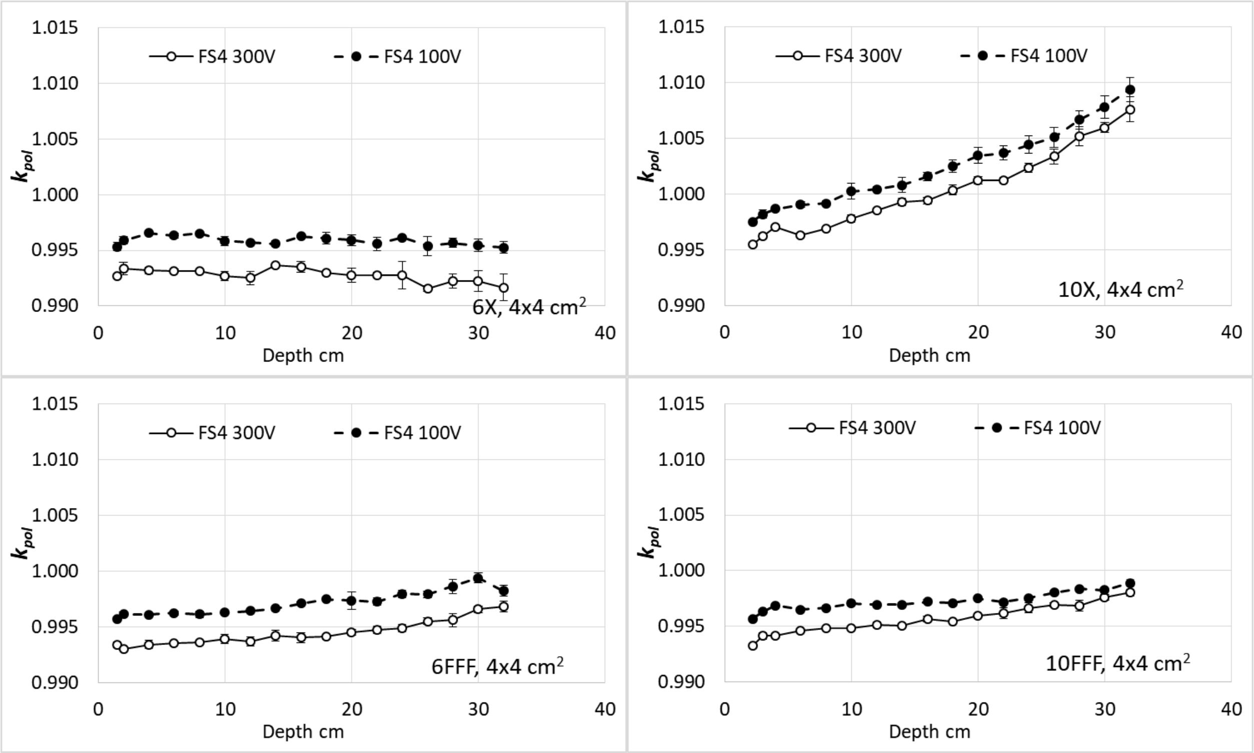

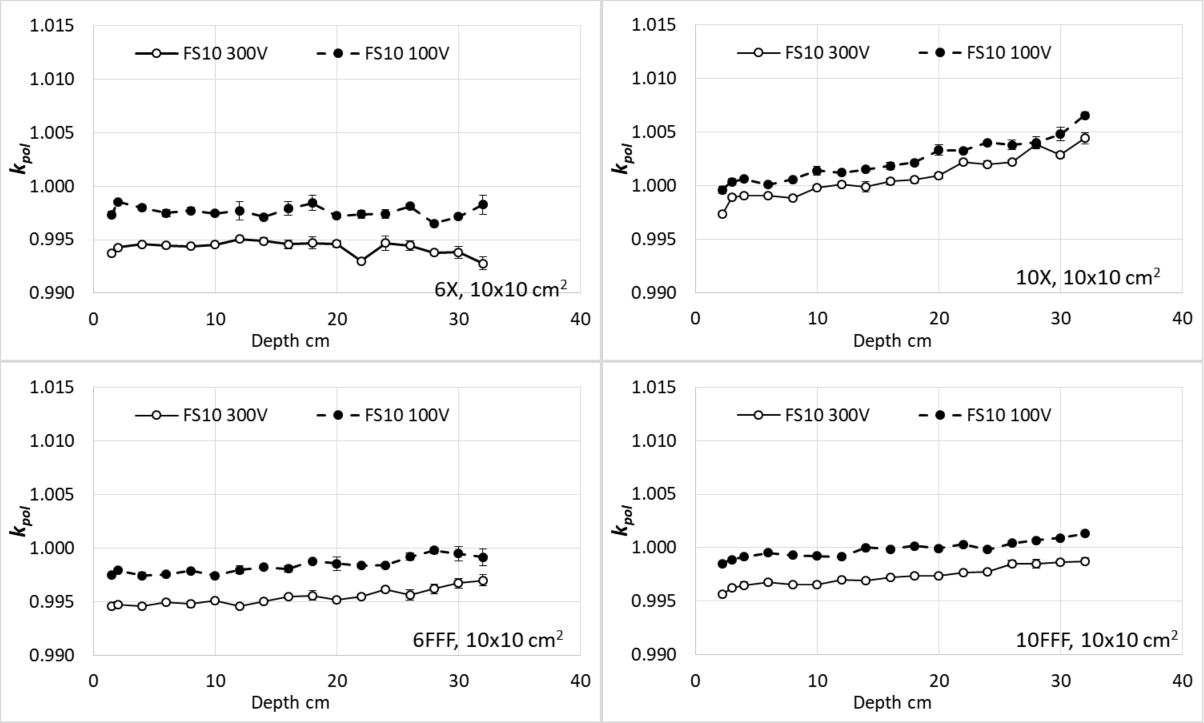
FS10 300V
FS10 100V
kpol
Depth cm
6X, 10x10 cm2
10X, 10x10 cm2
6FFF, 10x10 cm2
10FFF, 10x10 cm2
1.015
1.010
1.005
1.000
0.995
0.990
0
10
20
30
40

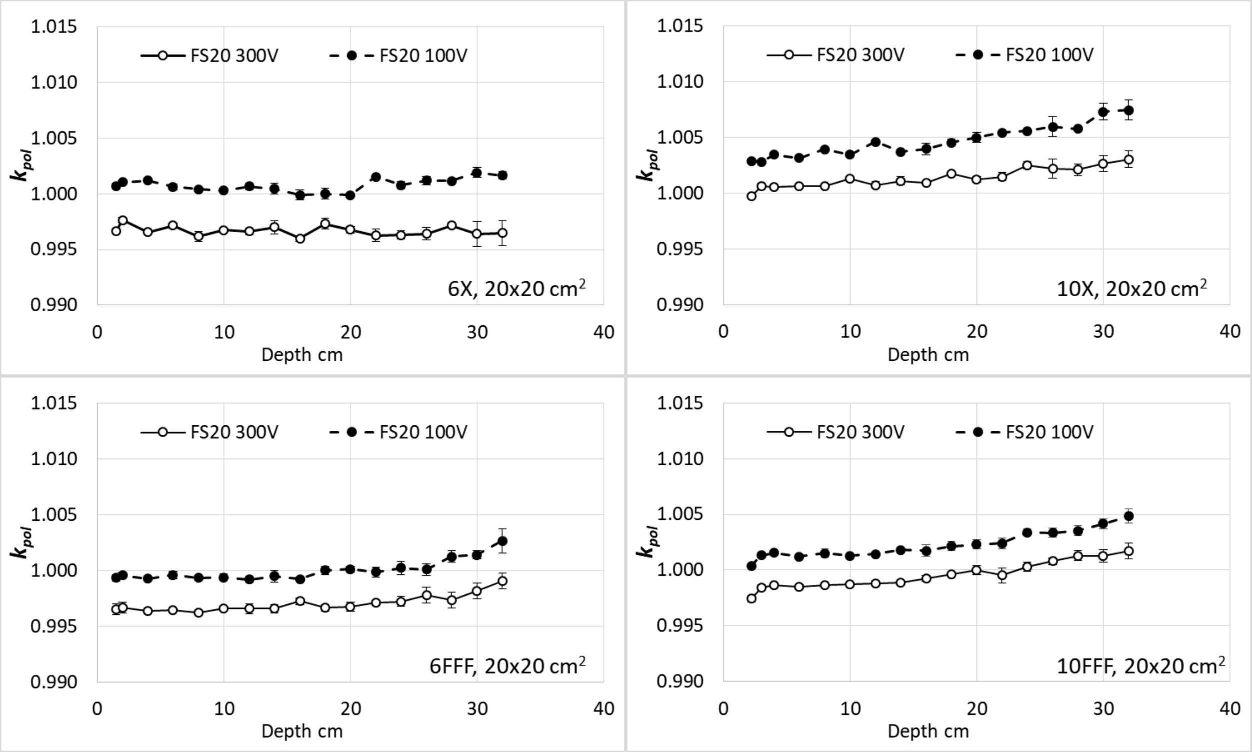
FS20 300V
FS20 100V
kpol
Depth cm
6X, 20x20 cm2
10X, 20x20 cm2
6FFF, 20x20 cm2
10FFF, 20x20 cm2
1.015
1.010
1.005
1.000
0.995
0.990
0
10
20
30
40

Figure 2s: k_s as a function of relative dose for 4x4, 10x10 and 20x20 cm^2 field size for all the analysed energies. The scale on the y-axis is the same in all plots. This Figure completes the data of Figure 2 in the main text, which reports the same results for the 40x40 cm^2 only.

(a) Semiflex-3D

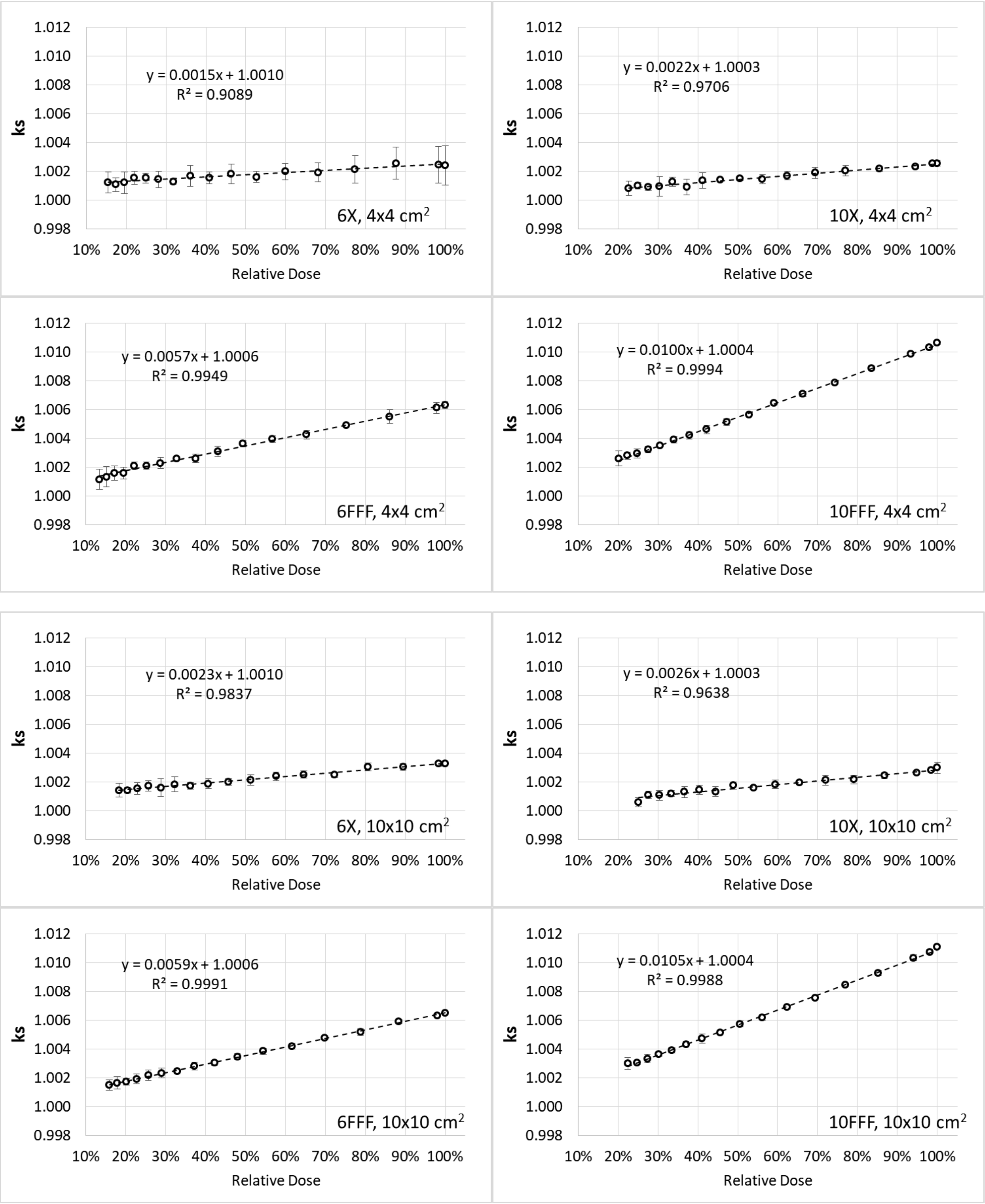

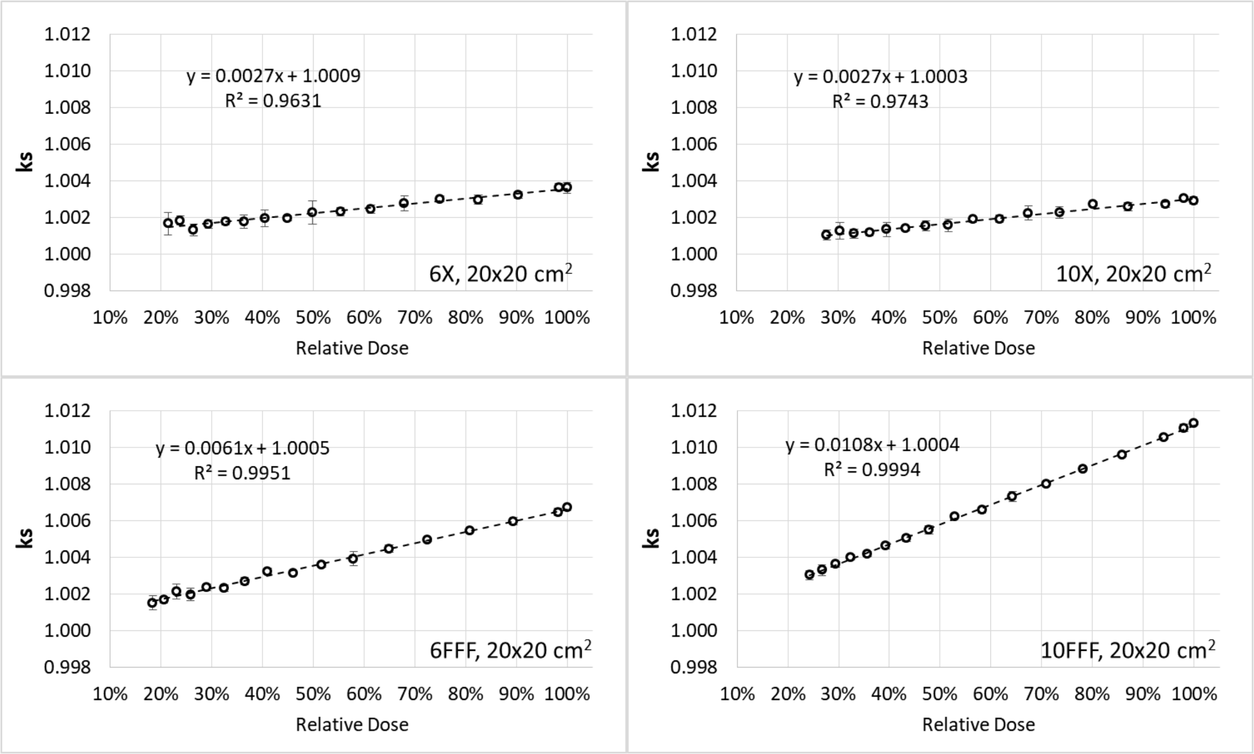

(b) PinPoint-3D

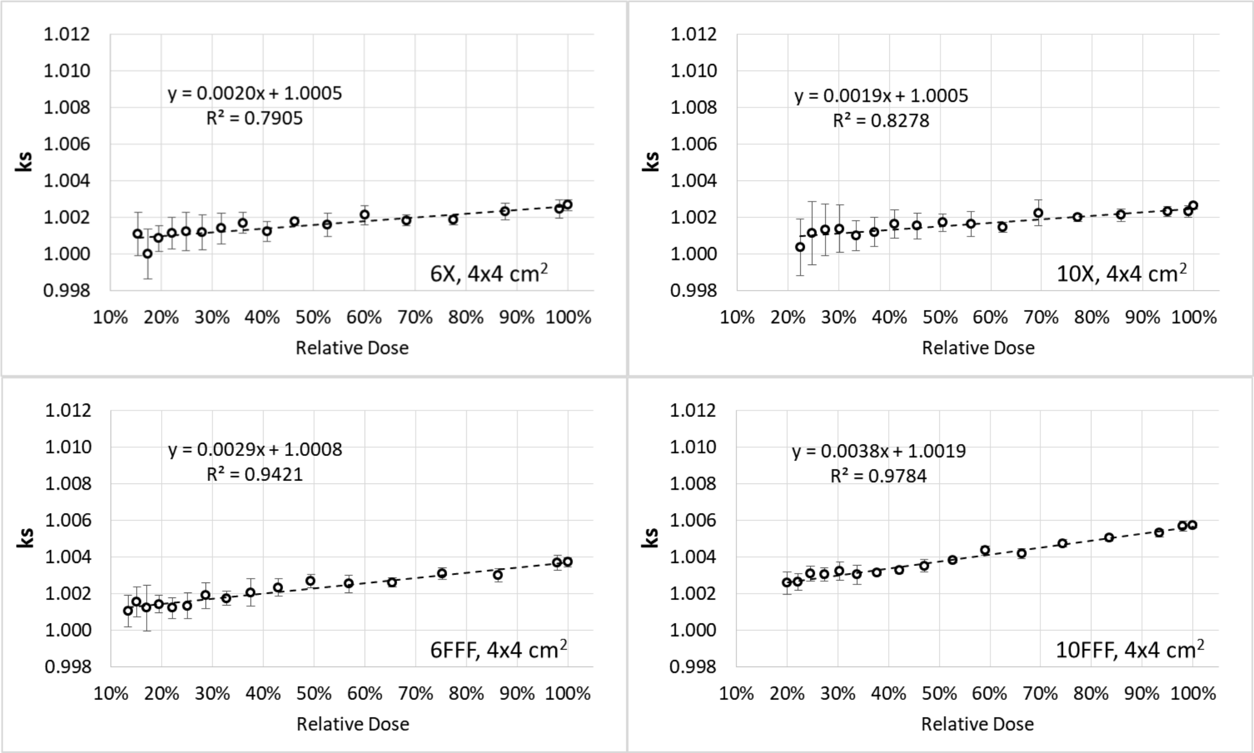

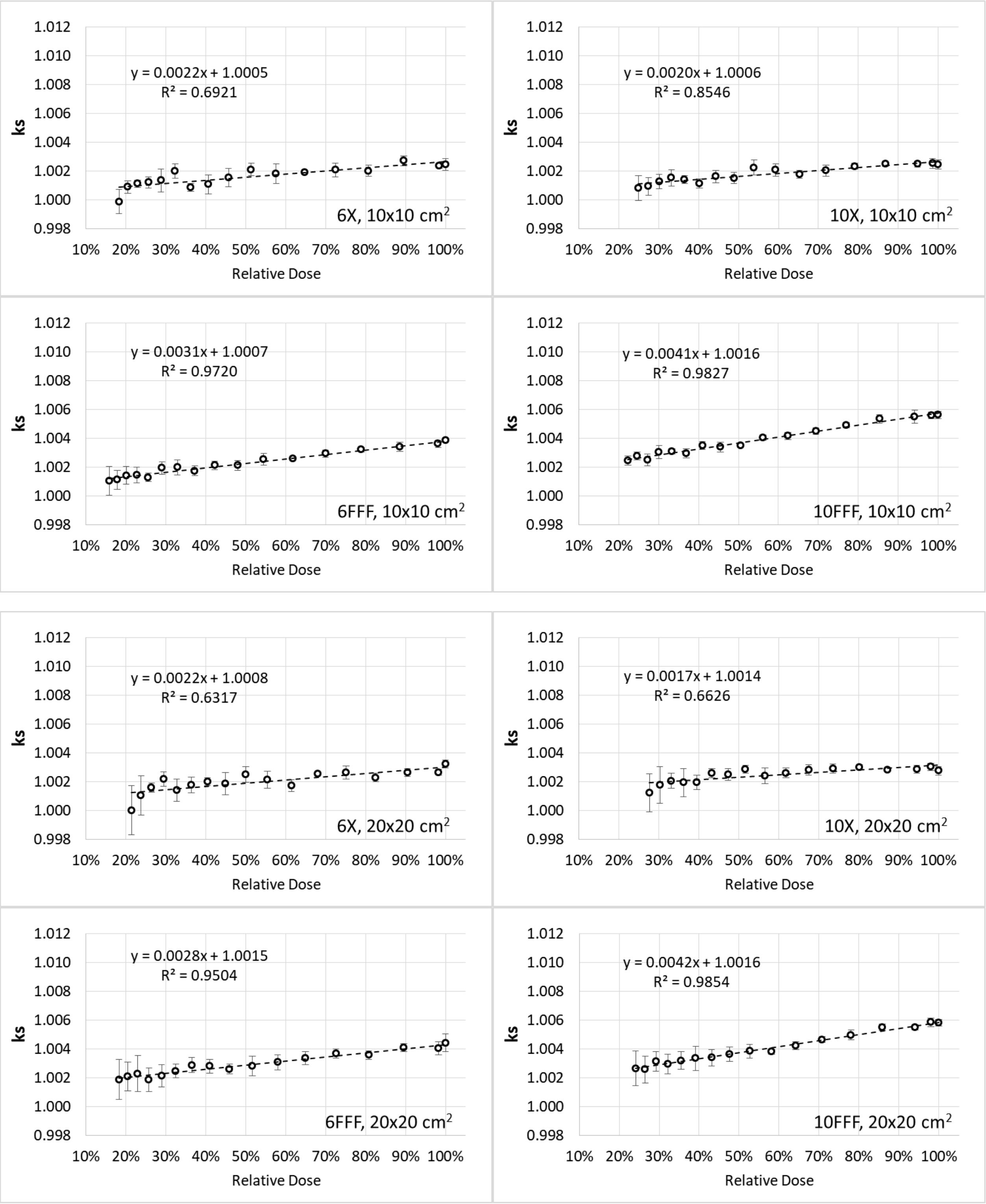

ks
Relative Dose
y = 0.0022x + 1.0005
R² = 0.6921
6X, 10x10 cm²
y = 0.0020x + 1.0006
R² = 0.8546
10X, 10x10 cm²
y = 0.0031x + 1.0007
R² = 0.9720
6FFF, 10x10 cm²
y = 0.0041x + 1.0016
R² = 0.9827
10FFF, 10x10 cm²
y = 0.0022x + 1.0008
R² = 0.6317
6X, 20x20 cm²
y = 0.0017x + 1.0014
R² = 0.6626
10X, 20x20 cm²
y = 0.0028x + 1.0015
R² = 0.9504
6FFF, 20x20 cm²
y = 0.0042x + 1.0016
R² = 0.9854
10FFF, 20x20 cm²

Figure 3s: Ratio between corrected and uncorrected PDD for the 4x4, 10x10 and 20x20 cm^2 field sizes for all the analysed energies. The fitting curve can be the correction factor to apply to the raw PDD scanned data acquired with the specific detector. The scale on the *y*-axis is the same in all plots. This Figure completes the data of Figure 4 in the main text, which reports the same results for the 40x40 cm^2 only.

(a) Semiflex-3D

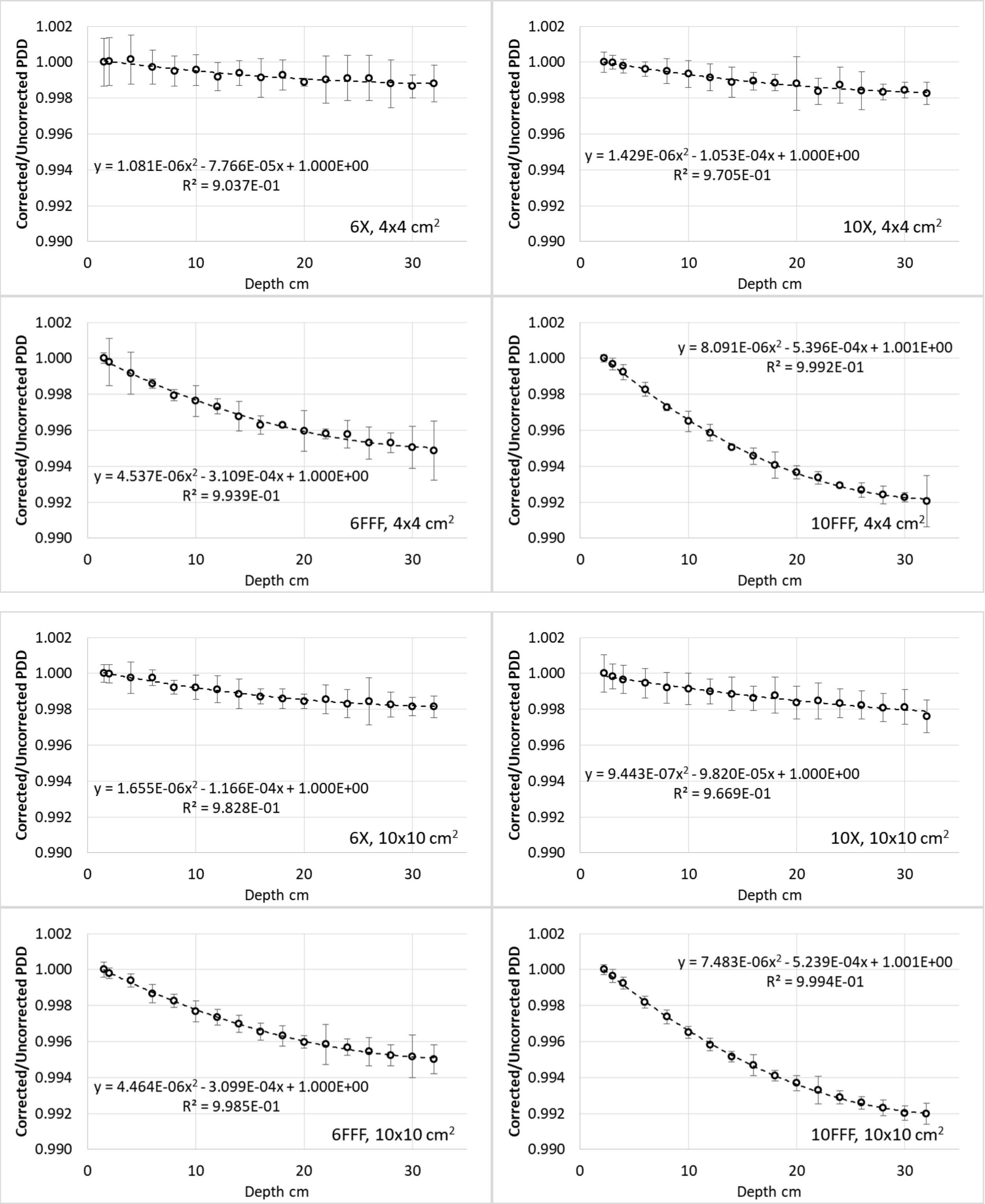

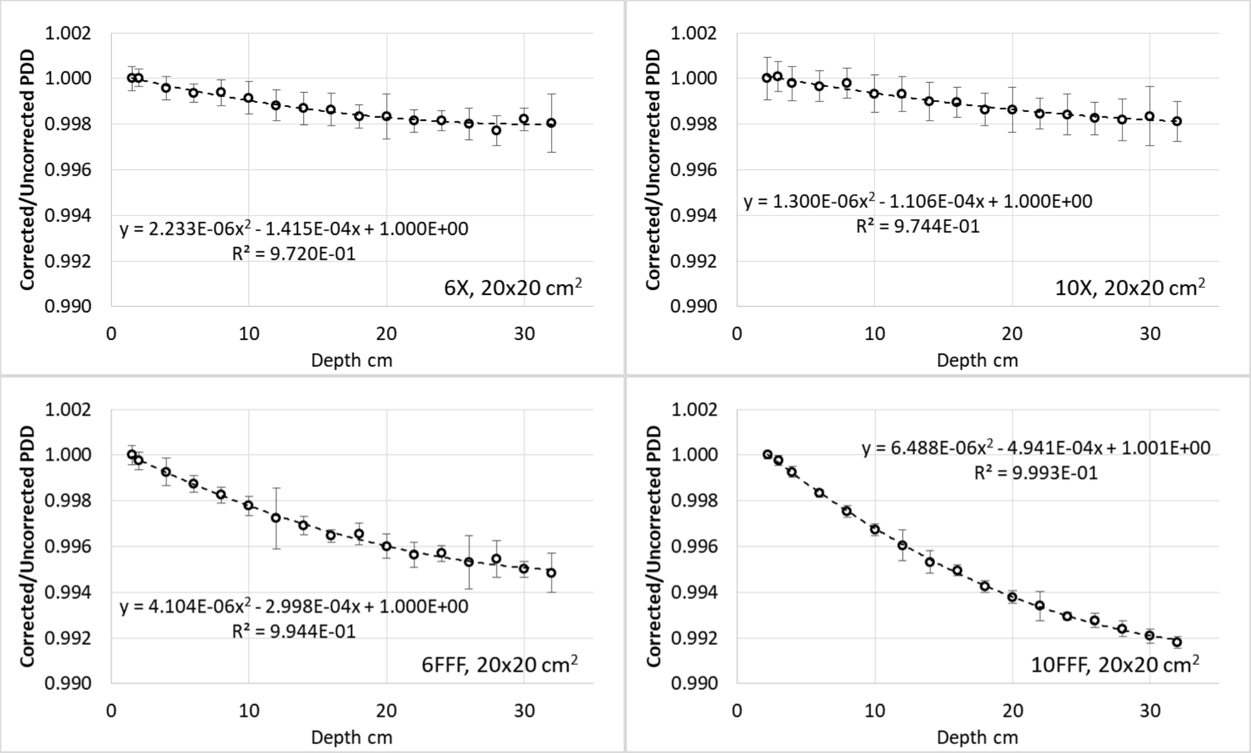

(b) PinPoint-3D

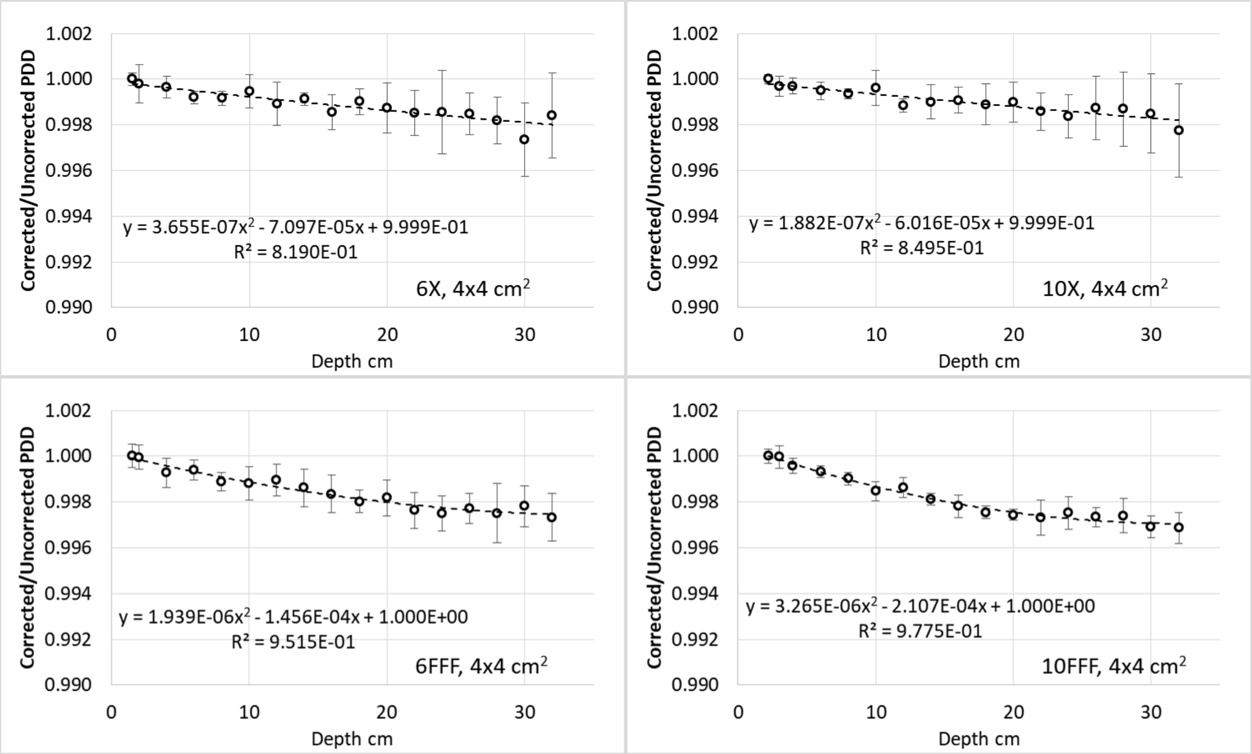

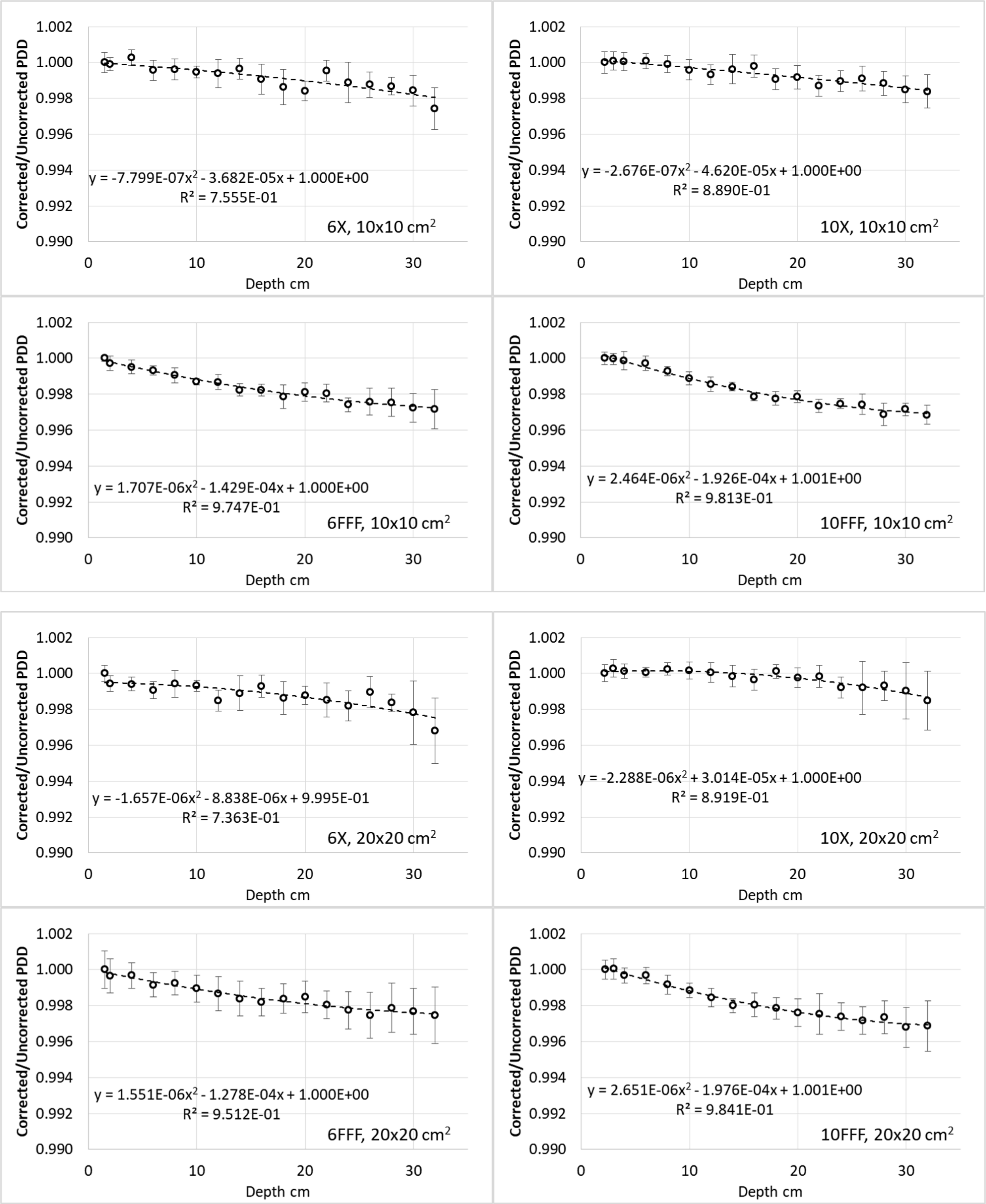
6X, 10x10 cm2
y = -7.799E-07x2 - 3.682E-05x + 1.000E+00
R² = 7.555E-01
10X, 10x10 cm2
y = -2.676E-07x2 - 4.620E-05x + 1.000E+00
R² = 8.890E-01
6FFF, 10x10 cm2
y = 1.707E-06x2 - 1.429E-04x + 1.000E+00
R² = 9.747E-01
10FFF, 10x10 cm2
y = 2.464E-06x2 - 1.926E-04x + 1.001E+00
R² = 9.813E-01
6X, 20x20 cm2
y = -1.657E-06x2 - 8.838E-06x + 9.995E-01
R² = 7.363E-01
10X, 20x20 cm2
y = -2.288E-06x2 + 3.014E-05x + 1.000E+00
R² = 8.919E-01
6FFF, 20x20 cm2
y = 1.551E-06x2 - 1.278E-04x + 1.000E+00
R² = 9.512E-01
10FFF, 20x20 cm2
y = 2.651E-06x2 - 1.976E-04x + 1.001E+00
R² = 9.841E-01
Corrected/Uncorrected PDD
Depth cm
1.002
1.000
0.998
0.996
0.994
0.992
0.990
0
10
20
30

Figure 4s: Results of the **Semiflex (type 31010**, 0125 cm^3 volume) ion chamber:

(a) k_{pol} as a function of depth for 10x10 and 40x40 cm^2 field size for all the analysed energies

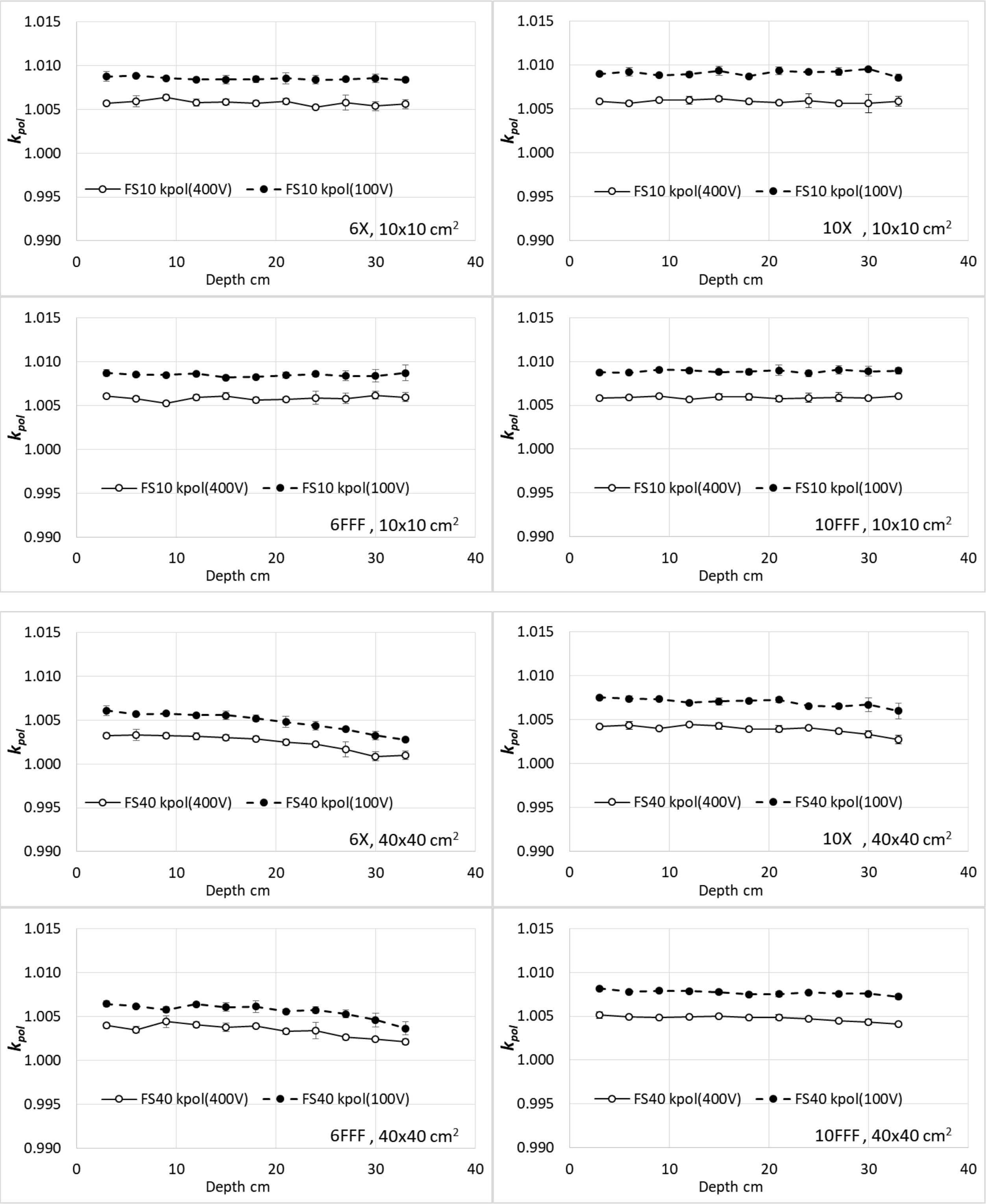

(b) k_s as a function of depth for 10x10 and 40x40 cm^2 field size for all the analysed energies

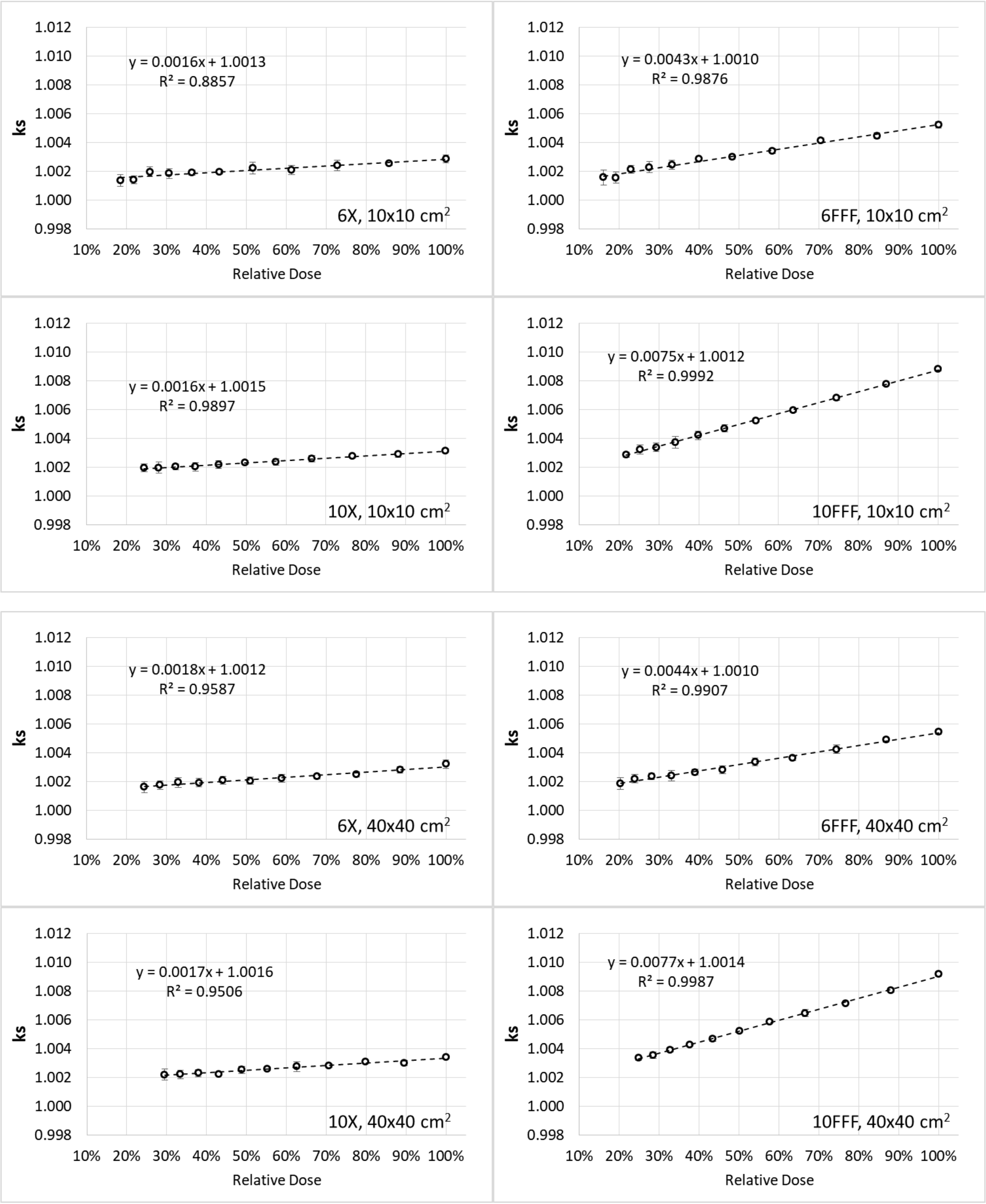

(c) Ratio between corrected and uncorrected PDD for the 10x10 cm^2 field sizes for all the analysed energies. The fitting curve can be the correction factor to apply to the raw PDD scanned data acquired with the specific detector. This Figure completes the data of Figure 5 in the main text, which reports the same results for the 40x40 cm^2.

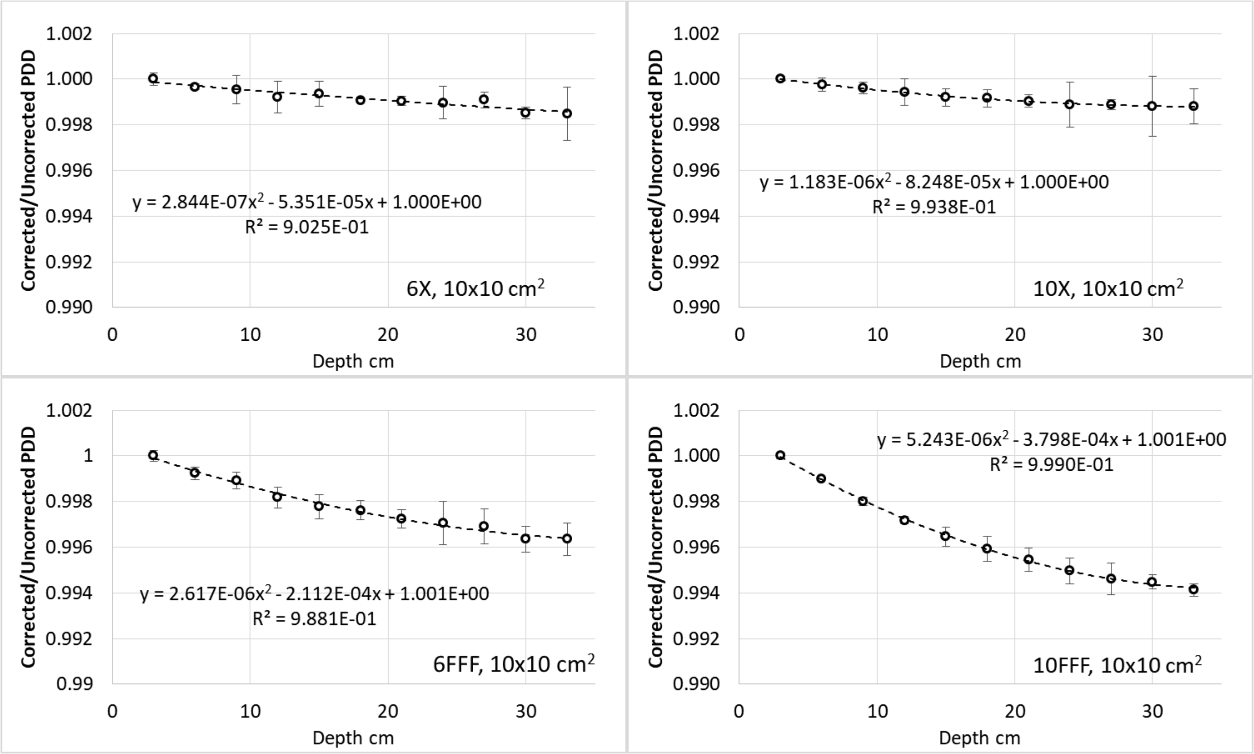

Supplement: Supplementary file 1 — SUPPORTING INFORMATION [file ACM2-26-e14495-s001.pdf]
